# Artificial Intelligence in Myopic Maculopathy: A Comprehensive Review of Identification, Classification, and Monitoring Using Diverse Imaging Modalities

**DOI:** 10.7759/cureus.78685

**Published:** 2025-02-07

**Authors:** Maria Varvara Kapetanaki, Eirini Maliagkani, Konstantinos Tyrlis, Ilias Georgalas

**Affiliations:** 1 1st University Department of Ophthalmology, "G. Gennimatas" General Hospital, National and Kapodistrian University of Athens, Athens, GRC

**Keywords:** artificial intelligence, deep learning, high myopia, machine learning, myopic macular degeneration, myopic maculopathy, ophthalmology, retinal imaging, review article

## Abstract

This review investigates the usefulness and effectiveness of artificial intelligence (AI) tools in the detection of myopic maculopathy lesions using traditional imaging techniques like fundus photography and optical coherence tomography (OCT). The role of machine learning (ML) and deep learning (DL) algorithms in the diagnosis, classification, and follow-up of highly myopic cases is discussed. A comprehensive analysis of articles published between 2018 and 2024 from PubMed, Science Direct-Elsevier, and Google Scholar identified 13 studies directly relevant to the topic. The majority of the studies were conducted in China and focused on patients with myopic macular degeneration and high myopia. The most popular AI algorithms included ResNet-18, ResNet-50, ResNet-101, DeepLabv3+ and DarkNet-19, Efficient Net (B0/B7), VOLO-D2, Efficient Former, ALFA-Mix+, and XGBoost. Reported statistical metrics ranged from 80% to 97.3% for accuracy, 80% to 99.8% for the area under the curve (AUC), 83.0% to 97.0% for sensitivity, 63.0% to 97.21% for specificity, and 0.8358 to 0.9880 for the kappa value. The findings reveal that AI models can play a supportive role in disease diagnosis, achieving performance metrics comparable to those of general ophthalmologists. Furthermore, the utilization of larger datasets of OCT and fundus images improves generalizability and diagnostic accuracy. The integration of multimodal imaging techniques, such as OCT, color fundus photographs, and ultra-wide field photographs, enhances diagnostic clinical value and enables more comprehensive disease monitoring.

## Introduction and background

Myopic maculopathy (MM) or myopic macular degeneration (MMD) is a frequent disease entity, appearing most often on the posterior fundus of highly myopic patients and constituting a major sight-threatening complication. High myopia is a widespread term referring to a significant negative refractive error (spherical equivalence less than -6.00 D), which is usually accompanied by axial length elongation (typically greater than 26.5 mm [1]. The primary pathological alterations occurring in the retina and the progressive nature of the disease underline the high clinical value of early intervention and treatment to achieve effective rehabilitation. The recent development of artificial intelligence (AI) techniques that are based on convolutional neural networks (CNNs) has opened new horizons for the application of deep learning methodologies that could significantly contribute (collaboratively with a general ophthalmologist’s potential) to the initial screening and management of these cases. CNNs are a type of artificial neural network (ANN), which is inspired by the biological brain’s architecture. These networks consist of an input layer that receives data, hidden layers that extract features, and a final layer that generates predictions or classifications based on extracted features. Several CNN models, including VGG, ResNet, DenseNet, and EfficientNet, have been developed to improve accuracy in analyzing medical images. CNNs have become essential tools in medical image analysis, widely used for classification, diagnosis, and various real-world applications. Their ability to process complex patterns and large datasets has significantly enhanced automated medical assessments, contributing to improved efficiency and accuracy in clinical decision-making [2].

This review presents the research results of several scientific studies carried out during the last decade in this field, whose primary role is the evaluation of the diagnostic value of these pioneer tools in everyday clinical practice via the utilization of a variety of statistical measurements and calculations, such as sensitivity, specificity, accuracy, area under the curve (AUC), and kappa value. The final stage of human-machine comparison, embedded in the majority of the studies analyzed, provides a non-negligible assessment of AI’s prospective integration in the diagnostic quiver and classification procedures of the disease’s spectrum. The satisfactory performance metrics of AI-based algorithms in the detection of the disease (when contrasted to a senior specialist) strongly confirm the usefulness or even necessity of this innovative technology in the recognition and follow-up of MM’s characteristic lesions. Nevertheless, extensive research and further processes regarding optimization and amelioration of these techniques are mandatory in order to reach full autonomy and potentially replace physicians’ role in the foreseeing future. The assistance of supplementary and more targeted experiments may provide a more thorough validation of external and internal testing methodologies and could allow AI techniques to reach the competitive skills of an expert and replace the workload of a general ophthalmologist in everyday clinical practice (full autonomy levels).

General characteristics concerning the classification and pathophysiology of the disease are essential for understanding the studies analyzed. Several longitudinal studies have been conducted to assess MM progression that is linked to increasing peripapillary atrophy. Initially, according to Hayashi et al., the early existence of lacquer cracks and staphyloma influence the evolution of atrophy in high myopia, indicating that these features are key points in monitoring the course of the disease [3]. These authors described a progression pattern whose first finding is a tessellated fundus (TF), which may evolve into diffuse atrophy or lacquer cracks - or less frequently - to myopic choroidal neovascularization [1].

Many classification systems have been proposed during the last decades, a brief grading from mild to severe (based on the International Photographic Classification and Grading System META-PM) is as follows: no myopic retinal degenerative lesions (Category 0), tessellated fundus (Category 1), Diffuse chorioretinal atrophy (Category 2), extramacular patchy chorioretinal atrophy (Category 3), and macular patchy chorioretinal atrophy (Category 4) [4].

Moreover, three kinds of “plus” lesions were described: Lacquer cracks, myopic choroidal neovascularization (CNV), and Fuchs’ spots. Based on this grading system, eyes of Category 2 or greater, with plus lesions, or with the existence of posterior staphyloma are defined as having pathologic myopia (PM). A more severe type of MM has more marked fundus alterations and a worse visual prognosis. Consequently, the timely recognition of these primary modifications in the posterior pole with the assistance of AI tools may prove to be mandatory to achieve more effective disease stratification and reduction of visual impairment and blindness in these patients [5].

As already mentioned, eyes with macular diffuse chorioretinal atrophy or with plus lesions were integrated into the severe MM group, while the eyes of Categories 0 and 1 or those with peripapillary diffuse chorioretinal atrophy were archived in the less severe type of the disorder. The new ATN classification examines all the aspects of this entity and describes atrophic (A0 - no myopic retinal lesions, A1 - tessellated fundus only, A2 - diffuse chorioretinal atrophy, A3 - patchy chorioretinal atrophy, A4 - complete macular atrophy), tractional (T0 - no macular schisis, T1 - inner or outer foveoschisis, T2 - inner and outer foveoschisis, T3 - foveal detachment, T4 - full-thickness macular hole, T5 - macular hole and retinal detachment) and neovascular (N0 - no mCNV, N1 - macular lacquer crack, N2a - active CNV, N2b - scar or Fuchs’ spot) effects to the sclera, choroid, and retina of highly myopic eyes [6]. After several studies, the pathology of myopic traction maculopathy has been interpreted not just as a group of different foveal and retinal changes in a tractional myopic environment but also as a single progressive disorder that encloses diverse stages of evolution [1].

All the above evidence suggests that MM is a highly complex disease that involves atrophic, tractional, and neovascular modifications in the posterior fundus of patients. The most essential factors driving MM’s progression are axial length elongation, expansion of the posterior eye wall, and staphyloma that may lead to irreversible photoreceptor loss and central visual impairment in adults [1].

The most representative features of the disease are cited as follows: 1) Retinal choroidal and scleral thinning are usually associated with the presence of posterior staphyloma formation [1]. 2. Tessellated fundus results from hypoplasia of the retinal pigment epithelium (RPE) surface and allows a clear illustration of the choroidal vascular network in color fundus images. It constitutes a feature that usually appears in younger cases with less myopia, shorter axial length (less than 26 mm), and absence of chorioretinal atrophy or staphyloma [4]. 3) Diffuse chorioretinal atrophy, is usually depicted as a yellowish-white area around the optic disc. On fluorescein angiography (FA), it is represented by milder hyperfluorescence in the late phase due to tissue staining. On OCT scans, significant thinning of the choroidal layer can be seen, whereas indocyanine green angiography (ICGA) shows significant reduction of the choroidal capillary network [4]. 4) Patchy chorioretinal atrophy that presents as grayish-white lesions in the macula or around the optic disc. The pathogenetic mechanism is a total absence of choriocapillaris and a complete destruction of the outer retina and RPE. In FA and ICGA imaging, a choroidal filling defect is observed around patchy atrophy that correlates with a loss of the entire thickness of the choroid, RPE, and outer retina on OCT scans. Recent swept-source OCT (SS-OCT) or histological studies reveal a discontinuity of Bruch membrane in the regions with patchy atrophy as well as in the peripapillary gamma zone. These defects rename the entity as “macular Bruch membrane rupture” [4]. 5) Macular atrophy refers to the state of occupation of the entire area of the posterior fundus including the foveal region (macula space), leading to central vision loss. In this case, the posterior pole obtains a “bare sclera” appearance with retrobulbar blood vessels notable through the thin transparent retinal tissue [4]. 6) Choroidal neovascularization is a major complication that occurs in areas of preserved choriocapillaris (higher vascular endothelial growth factor-VEGF levels in the aqueous humor) and is more frequent in eyes with less advanced staphylomas. The presence of a Fuchs’ spot (dark area in the macula) is indicative of a late-stage myopic CNV (regression of choroidal neovascularization) and is caused by hyperplasia and migration of RPE cells to the subretinal or intraretinal tissue. Chorioretinal atrophy will typically develop around the regressed membrane, leading to a poor visual outcome. Timely treatment with anti-VEGF injections is required in these cases to avoid severe visual loss [1,4]. 7) Myopic macular hole and myopic foveoschisis are induced by tractional changes in the myopic fundus. The development of a full-thickness macular hole (FTMH) in severe cases may provoke retinal detachment in these eyes attributed to persistent tractions over its margins [6]. 8) A dome-shaped macula is represented by the ventral configuration of the sclera and often related to a serous, non-evolving retinal detachment [1].

## Review

Search methodology

**Figure 1:**
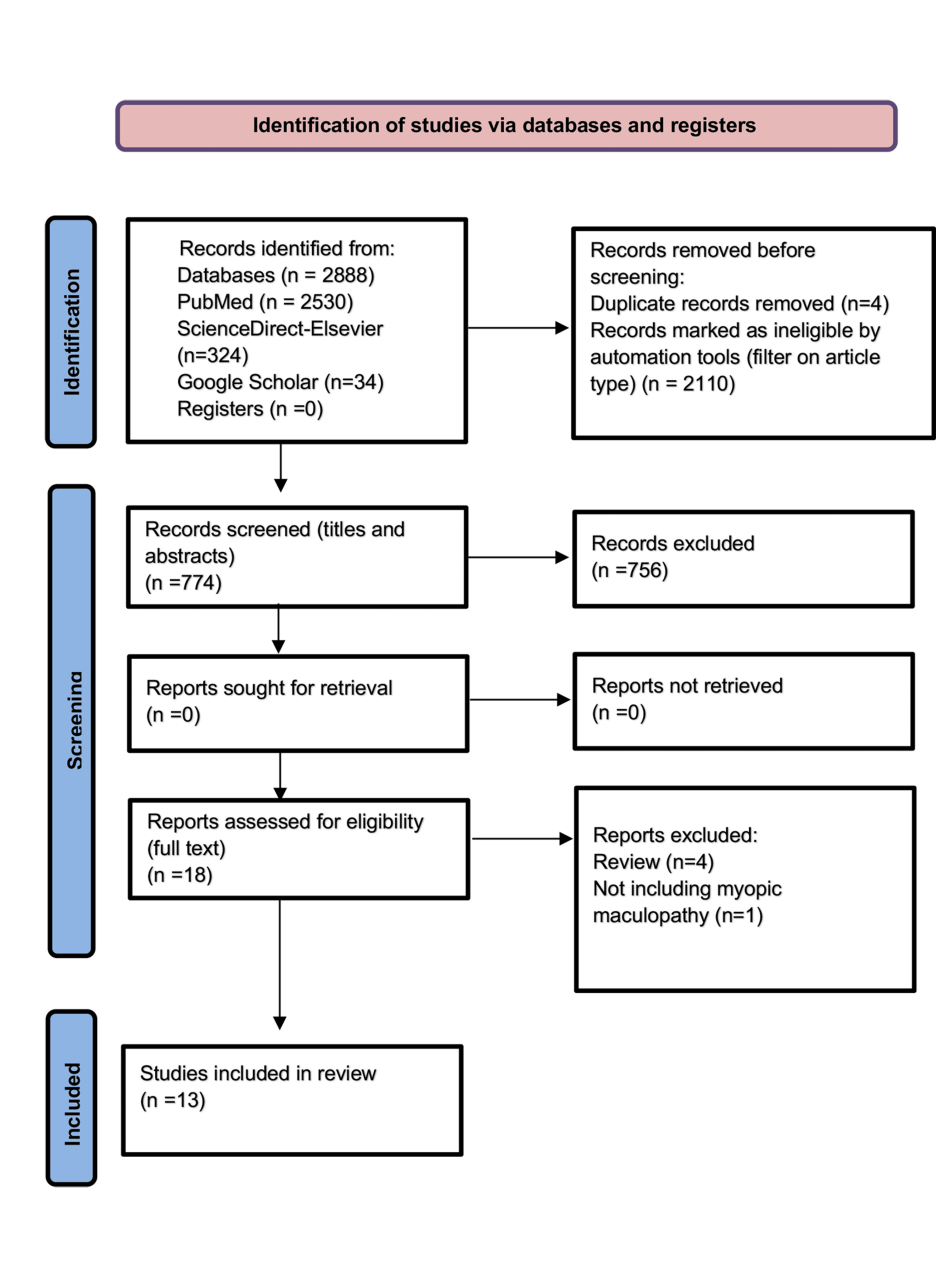
Preferred Reporting Items for Systematic Reviews and Meta-analyses (PRISMA) 2020 flow diagram.

This comprehensive review was developed through an online search of recent published studies (from 2018 to 2024) related to the subject of interest. The first step included the design of a specific algorithm containing keywords relevant to the topic that was applied across three different online platforms (PubMed, ScienceDirect-Elsevier, and Google Scholar), whose construction aimed to facilitate the searching procedure. Specific eligibility criteria were created to guide the selection of the most relevant studies. The inclusion criteria focused on studies investigating MM, using AI, machine learning (ML), or deep learning (DL) techniques, and reporting metrics like sensitivity, specificity, accuracy, and AUC, while the exclusion criteria eliminated non-human studies, reviews, abstracts, case reports, articles without full text, non-English publications, or studies outside the 2018-2024 timeframe. A total of 774 records were screened, evaluating titles and abstracts for relevance. After screening, 18 full-text studies were assessed for eligibility based on the predefined criteria, and 13 studies were included for final analysis. The selection process is summarized in the Preferred Reporting Items for Systematic Reviews and Meta-analyses (PRISMA) flow diagram (Figure 1), which provides a structured overview of study inclusion and exclusion steps [7].

Application of AI in MM

Following a chronological order, we will refer to four studies conducted during the year 2021 by several groups of scientists. The first one was carried out by Tan et al. in 2021 in Singapore who used a large stock of retinal fundus photographs (226,686 images) to assess the DL algorithm’s (ResNet-101, CNN architecture) potential in identifying, classifying, and screening MM cases. External testing was carried out on datasets from China, Taiwan, India, Russia, and the UK, whereas heatmaps were generated for the localization of lesions by applying the gradient × input method. The last stage consisted of performance evaluation via comparison against six retinal specialists based on a small number (~200 samples) of randomly selected images. Statistical analysis showed strong performance metrics across all datasets. For MMD, internal testing achieved an AUC of 0.978, sensitivity of 91.4%, and specificity of 94.2%, while external testing reported AUCs of 0.969-0.988, sensitivity of 96.8-98.4%, and specificity of 85.5-95.9%. For high myopia, internal testing showed an AUC of 0.978, sensitivity of 91.3%, and specificity of 94.5%, whereas external testing reported AUCs of 0.913-0.966, sensitivity of 85.3-97.8%, and specificity of 76.4-95.5%. Lastly, the algorithm overpassed six human expert graders in the recognition of the characteristic lesions, achieving similar error rates (97.5% sensitivity on external testing). The discoveries mentioned above indicate that AI-based models could substitute human potential, offering an effective alternative solution in the identification of cases at the highest risk for sight-threatening complications and providing a useful automatic screening tool in daily clinical practice [8].

The second study was carried out by Du et al. in 2021 (China), who examined 457 eyes with pathologic myopia lesions (CFP images). The radiomics analysis method was applied to identify features of the severe MM condition from the optic disc region. Receiver operating characteristic (ROC) curves were used as a measure of their performance in classifying the severe stage of the disease. The methodology consisted of the following elements: 1) delineation of the regions of interest (ROIs), 2) construction of a feature pool using an automatic extraction strategy, and 3) selection of discriminative features with the aid of correlation analysis and ML algorithm. The performance of the new image feature set in classifying severe MM was scored as 0.8263 on the validation dataset, whereas the performance of the clinic feature set was scored as 0.7925 AUC. The two categories were merged to form a union feature set that gained the number of 0.8358 on the validation set. The main findings of the research executed were the following: a) eight new image features were determined to show greater ability than clinic features in identifying severe ΜΜ lesions; b) an effective ΜL tool in extracting and deciphering various MM-related image features in an automatic manner was exploited; and c) deviations between peripapillary diffuse chorioretinal atrophy (PDCA) and macular diffuse chorioretinal atrophy (MDCA) were confirmed by both new image and clinic features. This discovery unraveled a more comprehensive view regarding the characteristic development and progression patterns of MM lesions [9].

**Figure 2:**
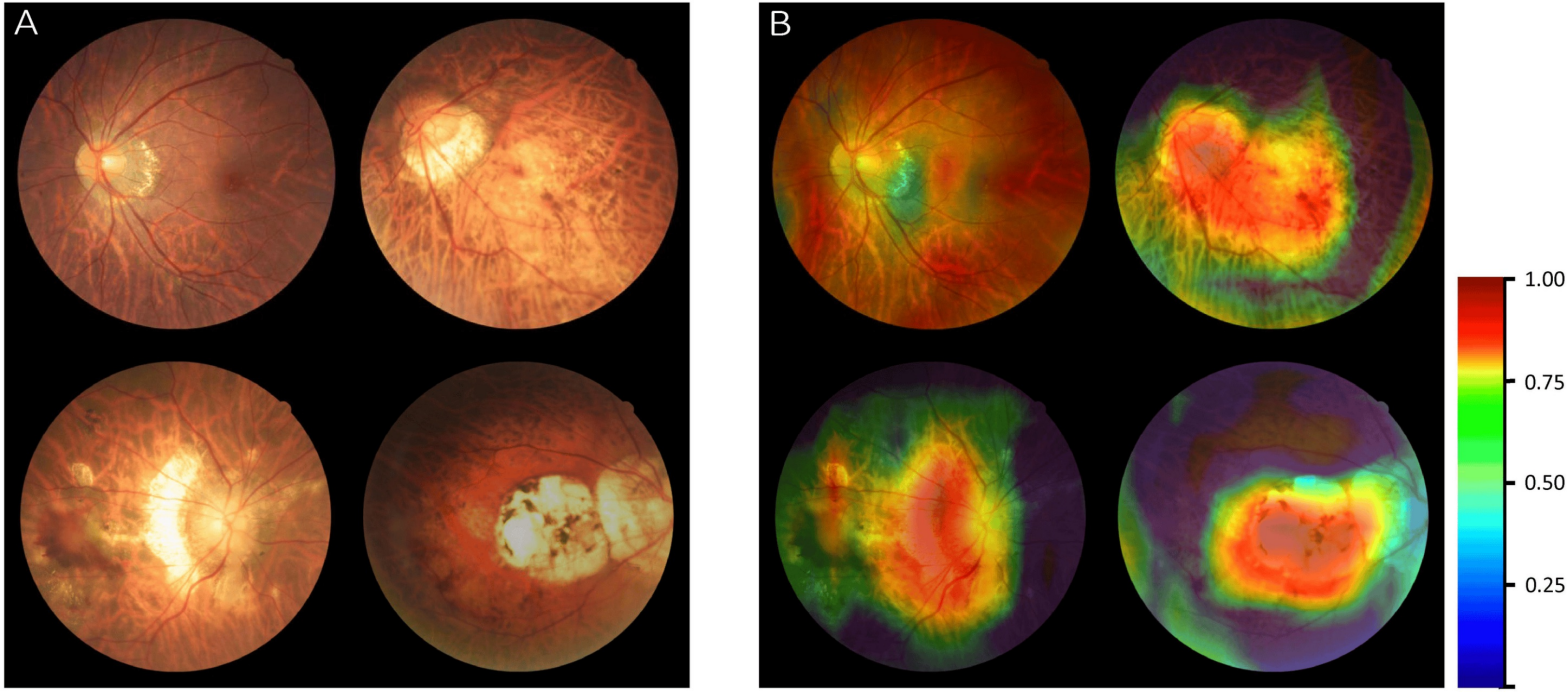
Visualization of algorithm II for classifying the category of myopic maculopathy (MM). (A) The original images of different MM (Category 1–Category 4). (B) Heatmap generated from deep features overlaid on the original images. The typical MM lesions were observed in the hot regions. Adapted from Lu et al. (2021), Frontiers in Cell and Developmental Biology, licensed under CC BY [10].

The third study conducted by Lu et al. in 2021 was the research product of a group of scientists who applied their methodology to 32,010 color retinal fundus images. This large stock of records was collected between July 2016 and January 2020 from the First Affiliated Hospital of the School of Medicine, Zhejiang University. These images were graded for training and cross-validation according to the META-PM classification system. Before the image analysis process, the scientists defined, annotated, and set a reference standard, deciding that fundus images of category 2 (diffuse chorioretinal atrophy) or worse that exhibited at least one of the “plus” lesions were considered to have pathologic myopia. A total of 20 ophthalmologists from three ophthalmic centers that achieved a kappa value ≥0.81 were selected to manually grade and annotate the images. The experts were separated into five groups (one senior specialist in each group) and the diagnosis that gained a unanimous vote by three graders of each team was set as the reference standard. After the gradable images were determined, algorithm I was generated according to the presence of pathologic myopia in each eye. On a second level, the gradable images were rendered an MM label category, a procedure that led to algorithm II creation. On a third level, experts localized “Plus” lesions within the image by drawing boxes and formed in this way Algorithm III. These three algorithms were used to develop two AI models (I and II); the first one contained Algorithm I and distinguished between non-pathologic myopia and pathologic myopia images, and the second consisted of Algorithms II and III and a logical analysis module based on the META-PM classification. Lastly, a comparison of their performances took place to obtain an optimized model for PM identification. Algorithms I and II were composed of a CNN architecture named ResNet18, whereas Algorithm III construction was based on a feature pyramid network (FPN), which contained faster region-based CNNs. The scientists executed external validation and expert-machine comparisons to assess the algorithms’ effectiveness more thoroughly, and with this aim, they recruited 1,000 images from three separate hospitals in Zhejiang Province. The images in the external validation dataset were simultaneously graded by the algorithms and two experts who did not participate in previous grading. Their comparison results served as tools for further performance quantification. The images misclassified by Algorithms I and II were further analyzed by a senior retinal specialist. A convolutional visualization layer was implanted at the end of Algorithm II that generated a heatmap and highlighted the strongly predictive regions on retinal fundus images. The following indexes were used to evaluate the algorithms’ performance: AUC (0.995), accuracy (0.973), specificity (0.981), sensitivity (0.939) for Algorithm I and macro-AUC (0.979), accuracy (0.967), and quadratic-weighted kappa (0.988) for Algorithm II. Algorithm III achieved an accuracy of 0.970 to 0.994 for recognizing plus lesions and an F1 score of 0.685 to 0.889 for identifying and localizing MM lesions. External validation contributed to the drawing of the following conclusions: a significant difference was observed between AI models and experts not only in identifying PM (P = 0.013) but also in distinguishing different MM lesions (P < 0.001). Nevertheless, the performance of the DL algorithms was almost equal to that of the experts. For instance, regarding PM identification, model 2 exhibited higher accuracy than the general ophthalmologist (96.9% vs. 96.1%), whereas for Algorithms II and III, the difference gap was within the spectrum of 3%. These statistical measurements confirm that the proposed AI models demonstrate satisfactory performance in a real-world setting and the algorithms’ application can facilitate diagnosis and screening in pathological myopia patients on a large scale in the future. A visualization of algorithm II for classifying the category of MM along with a heatmap illustration is presented in Figure 2 [10]. 

The fourth study was conducted by Ye et al. in 2021, who collaborated at the School of Ophthalmology and Eye Hospital at Wenzhou Medical University. The purpose of this investigation was to engineer a DL system using OCT images that could automatically screen and identify MM lesions. This AI model was developed with the aid of 2,342 OCT macular images acquired from 1,041 high myopia patients admitted to the Eye Hospital of Wenzhou Medical University from May 2016 to May 2020. The scientists trained five models that implemented the ResNest101 architecture to distinguish the following features: macular Bruch membrane (BM) defects, macular choroidal thinning, subretinal hyperreflective material, myopic traction maculopathy, and dome-shaped macula. With the aim of corresponding the AI system applied in real-world conditions, 450 images from 297 high myopia patients were chosen as an independent test dataset used for validation purposes. Each diagnosis was made via mutual consultation between two retina specialists and two ophthalmologists. The statistical analysis results for performance quantification regarding MM identification included an AUC measurement of ROC curves between 0.927 and 0.974 and sensitivity measurement equal to or greater than this of junior retinal specialists (56.16-99.73%) [11].

The final step of the research consisted of comparisons between the AI models and human retinal specialists. Each of the experts’ diagnoses was compared to a gold standard to evaluate the AI system's performance. For myopic traction, the maculopathy sensitivity of the AI system was calculated to be 92.80% and specificity 90.50%, whereas the corresponding metrics for junior specialists’ assessment were a sensitivity of 89.37% and specificity of 90.98%, and for senior specialists, it was 97.71% sensitivity and 96.93% specificity. Heatmaps were created not only for MM identification but also for macular BM defects (hallmarks of myopic CNV-related macular atrophy and patchy atrophy, indicating the absence of photoreceptors and lower visual acuity) and choroidal thinning that plays a pathogenetic role in the progression from normal fundus to diffuse atrophy. To summarize, the DL algorithm used by the researchers for the diagnosis and grading of MM was constructed based on a CNN architecture and achieved equal or better results than those of junior retinal specialists, since senior retinal specialists’ assessment maintained higher diagnostic levels [11].

Proceeding with the commentary and analysis, we will refer to four studies that took place during the next year (2022) by four different groups of researchers. The first one worth mentioning was conducted by Du et al. in 2022 in Japan and was built on 9,176 SS-OCT images gathered within the timeframe of October 2015 and March 2019 from patients with high myopia registered in the Advanced Clinical Center for Myopia in Tokyo. The scientists carried out their research through monitoring with periodic follow-up examinations of a total of 1,327 cases (2,400 highly myopic eyes, with a mean axial length close to 29.5 mm), whereas the ultimate purpose of the study was to develop a DL algorithm via training soft labels models with the aim of minimizing physicians’ uncertainty in diagnosing MM lesions. Nevertheless, the primary goal of the study was to compare and assess the differences between hard labels and soft labels in identifying myopic retinopathy through OCT image analysis but also to estimate the gap between human grading and these models in forecasting and classifying the disease. In total, 21 experts in myopia were involved in the research that examined the images for the presence of myopic neovascularization (MNV), myopic traction maculopathy (MTM), and dome-shaped macula (DSM), which are the most common alterations and the main reasons for vision deterioration in pathologic myopia eyes. After the image gathering and specialists’ assessment were completed, the stage of data processing followed. Model construction was divided into hard labels and soft labels, the first obeying the principle of majority overshadowing minority regarding the diagnosis results, whereas the second followed the idea of converting the labels into probabilities (calculation of possibility between different grading results). The training neural network used in the algorithms’ construction was Darknet-19. As a final step, model performance evaluation was held using statistical metrics such as kappa value (0.83 for MM, with P < 0.01). In comparison to the rest of the clinical entities examined, myopic traction maculopathy models (soft labels) presented a higher specificity and an area under the precision-recall curve (AUPR) greater than 0.876, with the calculation of an AUC close to 0.946. The DSM model achieved an AUC of 0.978 and AUPR of 0.653, whereas the MNV model achieved an AUC of 0.985 and AUPR of 0.908. Lastly, a remarkable finding of the study was extracted by the final comparison of the results between the AI models and the human graders. At first glance, the two methods showed similar consistency, but after a more thorough examination models trained by soft labels presented a slighter higher level of certainty compared to those trained by hard labels in MTM identification, whose uncertainty level was more approximate to that of the physicians. This conclusion was drawn using the kappa value as a calculation metric [12].

Another unique point of this research that needs to be underlined is that in real-life conditions, experts are often obliged to examine more than one OCT image to extract a definite diagnosis result regarding the entities mentioned before (MTM, MNV, DSM) since exact differentiation between them is not always achieved with a single scan evaluation. As a result, their final decision may adopt the vague outline of probabilities instead of a definite final answer, undermining in this way timely intervention in the emergence of sight-threatening complications. On the other hand, DL methodologies are built on the opposite logic of categorizing diseases into discriminate groups, thus providing an effective means of diminishing this ambiguity. Therefore, the innovative perspectives proposed by the authors (hard and soft labels) constitute a thorough attempt to clarify/resolve the uncertainty behind each individual diagnosis by discovering soft labels’ potential to mimic real-life scenarios and by confirming their superiority in drawing more accurate diagnostic results. Finally, two important advantages of the studies worth citing are the following: a) the effectiveness of the models trained by soft labels in incorporating the scientists’ uncertainty during the conclusions drawing process, improving in this manner performance in image recognition by creating a simulation of real-life conclusions on OCT scans decoding, and b) the utilization of hospital-based database that contributed to the generalizability of the study carried out [12].

Continuing, He et al. in 2022 used DL methodologies that were based on transfer learning (TL) to formulate an automated AI grading system that could recognize different stages of MM guided by OCT image results. Briefly, the scientists proceeded in this retrospective study by exploiting a stock of 3,400 macular OCT photographs acquired from 2,866 myopic patients. The ATN classification system functioned as a leverage for the development and training of two separate Algorithms (A and B). Although both served to identify targeted MM lesions, Algorithm B included the complementary element of TL and therefore demonstrated higher performance with a macro-AUC calculation of 0.986, accuracy of 96.4%, and kappa value of 0.986. In the test dataset, the algorithm achieved an AUC of 0.986, an accuracy of 96.04%, and a quadratic-weighted kappa of 0.940. On the external validation dataset, its performance remained strong, with an AUC of 0.938, an accuracy of 90.6%, and a quadratic-weighted kappa of 0.897. As far as human-machine comparison testing is concerned, the algorithms’ results appeared to be inferior to retinal specialists but equal to ordinary ophthalmologists in recognizing various posterior pole alterations. The ResNet-18 architecture was unraveled to derive meaningful output information on macular schisis recognition. Finally, a visual heatmap analysis of Algorithm B was carried out that highlighted with hot colors the most determining areas for classification of the disease on the original image [13].

To continue with the analysis process, we will focus on two studies that were based on color fundus photographs and were published almost simultaneously (during the year 2022) by two different groups of experts. The first one was conducted by Tang et al. in 2022 and focused on the construction of a DL model suitable for the diagnosis, identification, and segmentation of myopia-related lesions. Four specialists were assigned to annotate and classify the images with the use of DeepLabv3 + and ResNet-50 networks, respectively. On the next scale, the two models were combined to generate an integrated co-decision system for classification and segmentation analysis. In total, 1,395 CFPs were gathered from 895 patients, whereas the final statistical measurements for the evaluation of the model were an accuracy of 0.9370, quadratic-weighted κ coefficient of 0.9651, and AUC of 0.9980 in diagnosing pathologic myopia. These findings (demonstration of sufficient performance results) translate into the assumption that the application of the aforementioned automated techniques could successfully not only grade and diagnose but also monitor and follow-up advancement of the disease’s alterations on a clinical level, facilitating the task of ophthalmologists [14].

The second study worth commenting on was conducted by Li et al. in 2022 and used a deep CNN (D-CNN) system to recognize the absence of MM, tessellated fundus, and pathologic myopia. An amount of 57,148 images were graded by four ophthalmologists and analyzed using internal and external datasets to evaluate the model’s performance. The system achieved its best performance metrics in the SDEH dataset with a sensitivity of 93.3%, specificity of 99.6%, and AUC of 0.998 for PM recognition, whereas the corresponding measurements for TF identification were 98.8% for sensitivity, 95.6% for specificity, and 0.986 for AUC parameter. These numbers confirm the system’s robust potential in differentiating a variety of MM lesions on a large scale and significantly raise the probabilities of a real-life application of this innovative approach in the next decades [15].

Advancing the search to the next year’s investigations, we discover the study conducted by Mao et al. in 2023 after recruiting 203 subjects (317 eyes with high myopia examined) during July 2020 and January 2021 for evaluation at the Eye Hospital of Wenzhou Medical University in China. The analysis was based on UWF retinal imaging examination (green laser images) and the parameters measured were vessel angle, fractal dimension, vascular density, and number of vascular branches. The research included 61 healthy control subjects (104 normal eyes examined) to carry out comparisons. First, the images were classified into five categories (C0-C4) according to the META-PM grading system and then TL technology (RU-net network) was used to depict retinal vessels fundus distribution in different degrees of high myopia samples. Second, statistical analysis calculations were made, and correlations regarding axial length, best corrected visual acuity (BCVA), age, and vascular characteristics were performed with the assistance of Pearson tests (P < 0.05), whereas paired samples were evaluated with t-tests application. Segmentation analysis of UWF images in collaboration with a DL system utilization exhibited satisfactory performance metrics with an accuracy of 98.24%, sensitivity of 71.42%, specificity of 99.37%, and precision of 73.68%. Along with the disease’s evolution (increased severity of MM and eyeball elongation), vessel density and angles decreased, and fewer vascular branches were noted (these differences were also observed in comparison with healthy individuals, and they constitute statistically significant measurements; P < 0.001). Lastly, an association pattern between these retinal vascular morphology parameters and age (older), BCVA (worse), and axial length (longer) were observed [16].

**Figure 3:**
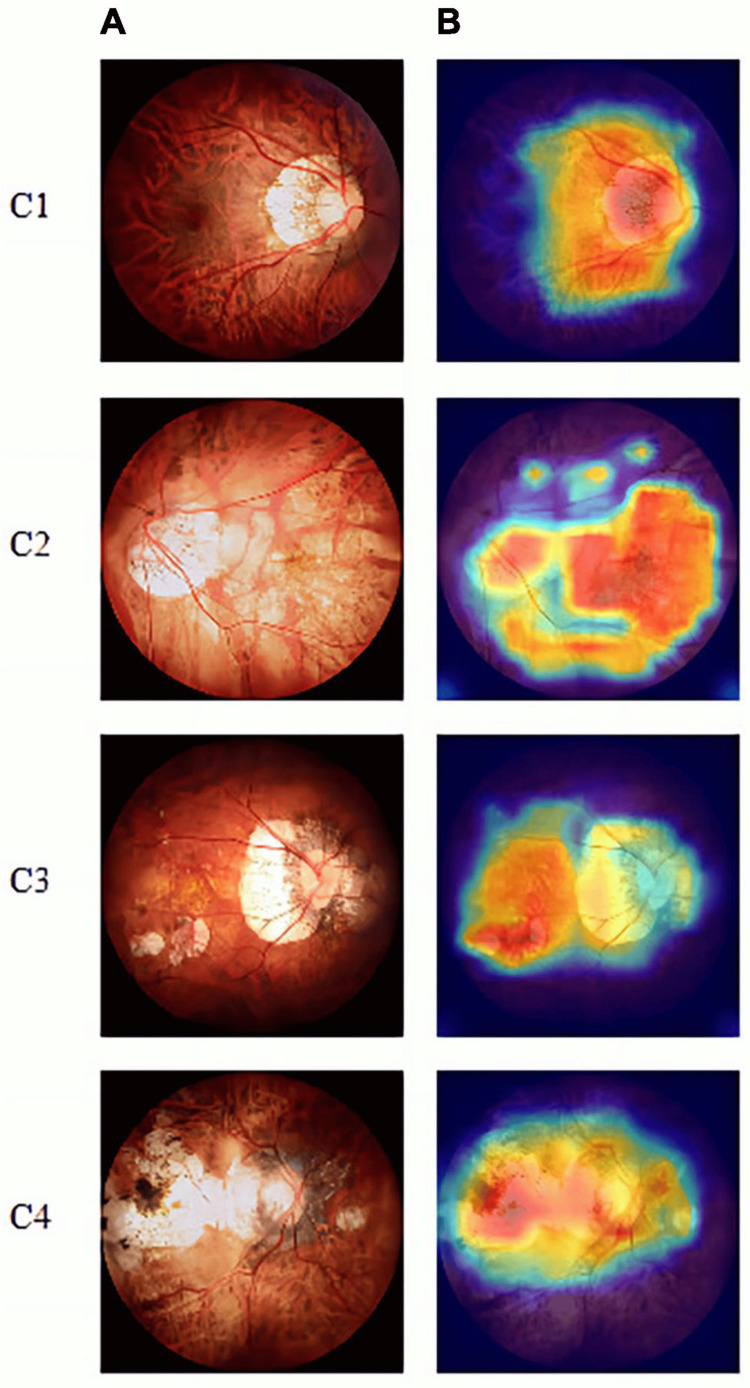
Visualization-based diagnosis by the VOLO-D2 model. (A) Original image. (B) Grad-CAM. Grad-CAM shows that the model focused on the myopic maculopathy area. From left to right, we can see the original fundus images of the four lesions along with the corresponding gradient activation heat maps. Grad-CAM, gradient-weighted class activation mapping. Adapted from Wan et al. (2023), Frontiers in Computational Neuroscience, licensed under CC BY [17].

Wan et al. in 2023 proposed an MM grading model for the automatic screening of lesions by examining a satisfactory number of retinal fundus images (1,750 photographs). The five-category models were trained with Vision Out Looker for Visual Recognition (VOLO), EfficientNetV2, and ResNet50. Among a large number of color fundus images initially gathered from the Eye Hospital of Nanjing Medical University, 174 fundus images were finally chosen for external testing. The diagnostic results were assessed using evaluation metrics such as sensitivity, specificity, negative predictive value (NPV), positive predictive value (PPV), AUC, kappa value, accuracy, and ROC curve. The accuracy of the VOLO-D2 model was measured to be 96.60% with a kappa-value of 95.60%, proving its effectiveness in identifying pathological macular lesions. For the diagnosis of images classified as C0 (absence of myopic lesions) the model achieved a performance of 100% in all metrics. Regarding the leopard appearance of the fundus (C1), the model’s sensitivity reached the percentage of 96.43%, NPV was 98.33%, whereas specificity and PPV were rendered with the highest ranking of 100%. As for the diffuse chorioretinal atrophy subgroup (C2), the following metrics were recorded: AUC 97.73%, sensitivity 96.88%, specificity 98.59%, PPV 93.94%, and NPV 99.29%. Relatively similar measurements (but a little lower) were observed for categories C3 (patchy chorio-retinal atrophy) and C4 (macular atrophy), with a performance of 100% (sensitivity) and 98.10% (specificity) for the last one. In addition, the scientists generated maps that were based on gradient-weighted class activation (Grad-CAM), to visualize the diagnosis extracted by the VOLO-D2 model regarding MM lesions recognition. EfficientNetV2-S demonstrated relatively lower performance in the diagnosis of all categories, with a sensitivity >89% and a higher consistency rate. Finally, the ResNet50 model achieved low sensitivity in macular atrophy identification and scored better results in non-myopic retinal degenerative and leopard fundus lesions. Based on the statistics above, these models can prove to be useful tools in the initial automatic screening of MM lesions. A visualization-based diagnosis by the VOLO-D2 model along with a Grad-CAM illustration is presented in Figure 3 [17].

Zhu et al., in 2023, ran simulations on classifying high MM lesions with the use of advanced DL models and a complete training set of 4,252 images. The primary aim of the study was to optimize the ALFA-Mix active learning algorithm and achieve superior grading results and enhanced model performance by combining it with EfficientFormer (an effective solution when labeled data are limited). A short description of the model’s operation is as follows: In the first stage feature extraction takes place from unlabeled and labeled images, which are then mixed to elicit predictions. In the second stage, the algorithm identifies the most representative samples with high information content and submits them to experts for annotation. In this manner, the cost required for expert annotation in a large dataset is reduced, maintaining high accuracy levels. This combination reached high evaluation metrics with an accuracy of 0.8964, sensitivity of 0.8643, specificity of 0.9721, and kappa value of 0.8537. The scientists introduced with their investigations an innovative approach that offers a convenient alternative in the analysis and diagnosis of high MM cases. This is mainly due to the algorithm's ability to find the golden ratio between uncertainty and variability, improving disease classification accuracy [18].

**Figure 4:**
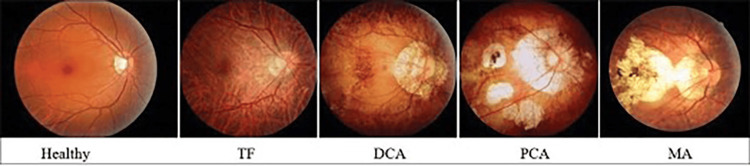
Images of a healthy fundus and the four degrees of myopic maculopathy. Adapted from Zheng et al. (2023), Indian Journal of Ophthalmology, licensed under CC BY-NC-SA 4.0 [19].

Zheng et al., in 2024, assessed 1,199 color fundus photographs in Shenzhen Eye Hospital (China) to develop an MM grading system using DL methods (Efficient Net-Bo) and struggle with the problem of delayed diagnosis and classification of different MM categories. The application of this AI scaling method facilitated the identification and distinguishing between the different degrees of MM and normal fundus images (Figure 4) [19].

Accurate diagnostic results of the disease were obtained through examination of the photographs by two ophthalmologists, but the contribution of a third specialist was mandatory in case of uncertainty. IBM SPSS Statistics (IBM Corp., Armonk, NY) was used to derive conclusions regarding the sensitivity of the model for tessellated fundus (96.86%), diffuse chorioretinal atrophy (75.98%), patchy chorioretinal atrophy (64.67%), and macular atrophy (88.75%). The specificity measurement for all four types of MMD was >93%. The kappa value of the EfficientNet-B0 model was calculated to be close to 84%, an indicator of a strong diagnostic consistency. The study also trained EfficienNett-B1 to B7, VGG16, and ResNet50 models, which demonstrated lower diagnostic results compared to the EfficientNet-B0 model. It must be noted that all different types of models achieved 100% accuracy in diagnosing healthy color fundus photographs. In total, 10 separate AI models (EfficientNet-B0-B7, VGG16, and ResNet50) were used to extract evaluation index results concerning the disease’s classification [19].

Based on these observations, the researchers arrived at the conclusion that the preliminary screening of MM by applying the algorithms above can result in an effective scaling system for disease classification, improving responsiveness and delaying vision impairment in these patients. The EfficientNet-based MM grading models demonstrated high sensitivity and specificity in diagnosing the disease’s progression by recognizing healthy fundus and four types of MM in color fundus photographs [19].

Finally, a more recent study published in June 2024 was written by Chen et al. and took place in the Zhongshan Ophthalmic Center in China. Its purpose was to forecast MM’s progression by developing and validating an interpretable ML algorithm. In total, 660 patients with high myopia were included in the research, which were followed up for 10.9 years on average. Between them, a relatively low percentage (20.2%, 133 cases) exhibited evolution of the disease in the following manners: newly emerging alterations (51.9%, 69 cases), arising of patchy atrophy out of diffuse atrophy (8.3%, 11 cases), spreading of lesions (40.6%, 54 cases), and reporting of plus signs’ development (6.8%, nine cases). During the monitoring period, a series of retinal images were gathered by the patients examined that allowed determination of progression patterns of the disease and formation of a risk assessment system. Two ophthalmologists and one retinal specialist graded fundus photographs captured according to the META-PM classification system, whereas OCT imaging served as an auxiliary tool in case of suspicion of CNV development in color fundus images. The ML algorithms applied to perform the investigation were indicatively XGBoost, logistic regression, random forest, light GBM, and neural network. Among them, the XGBoost algorithm presented the highest detection capability with accuracy close to 0.84, sensitivity of 0.83, specificity of 0.85, and F1 score of approximately 0.84, confirming its superiority in internal testing. In the external validation dataset, the XGBoost model achieved an AUC of 0.80, a sensitivity of 97.0%, a specificity of 63.0%, and an F1 score of 83.0%. These findings highlight the model’s generalizability and its potential to predict MMD progression in independent cohorts. After completing the validation phase of the models on both the internal and external datasets, statistical analysis was conducted via comparisons with t-tests and Mann-Whitney test measurements for continuous data or chi-square tests and Fisher exact test measurements for categorical data. As a fact, a p-value of less than 0.05% was statistically significant [20].

To summarize, this study is based on long-term data and observations to predict MMD progression over a decade in high myopia cases. The methodology unfolded via these algorithms’ deployment displayed high accuracy and robust clinical value in estimating future MMD traits. A major finding that must be highlighted is that the sub-foveal choroidal thickness (SBCT) parameter that derives from SS-OCT calculations is the most variable factor for forecasting the evolution of the disease. A satisfactory explanation for this phenomenon could be that prolonged choroidal ischemia due to increased axial length contributes to MM development. Another point that must be highlighted is the unique characteristic of the study to include a wide range of ages, permitting in this manner detection of the sight-threatening complications of the disease in younger age groups (e.g., adolescents). It is an element that holds precious clinical value given the rapid progression of the disease in this group of patients and emphasizes the need for early screening and intervention in a primary stage to avoid dangerous complications. The arguments above confirm and reinforce the urgency for the identification of changes on a first scale to ensure maximum healthcare benefits for this vulnerable population category [20]. 

A summary representation of the analyzed studies’ results is provided in Table 1.

**Table 1:** Summary of the analyzed studies and their reported performance metrics. CFP: color fundus photography, OCT: optical coherence tomography, SS-OCT: swept-source optical coherence tomography, UWF: ultra-wide-field, ResNet: residual network, CNN: convolutional neural network, FPN: feature pyramid network, DarkNet: dark network, DCNN-DS: dual-stream deep convolutional neural network, EfficientNet: efficient neural network, RU-net: reinforced U-Net, VOLO: Vision Outlooker for Visual Recognition, Alfa-Mix+: Active Learning by FeAture Mixing+, DenseNet: dense network, VGG: Visual Geometry Group, XGBoost: extreme gradient boosting, MMD: myopic macular degeneration, AUC: area under the curve, MTM: myopic traction maculopathy, DSM: dome-shaped macula, MNV: myopic neovascularization, PM: pathologic myopia, TF: tessellated fundus, SDEH: Shandong Eye Hospital, QDEH: Qingdao Eye Hospital, AUROC: area under the receiver operating characteristic curve, AUPRC: area under the precision-recall curve

Author	Country	Diseases included	Problem type	No. of images/ eyes	Imaging modality	AI algorithms	AI model performance
Tan et al. (2021) [8]	Singapore, China, Taiwan, India, Russia, UK	Myopic macular degeneration, high myopia	Detection, risk stratification, screening	226,686 images	Retinal photography	ResNet-101	For MMD (internal test): AUC: 0.978, sensitivity: 91.4%, specificity: 94.2%. For MMD (external test): AUC: 0.969-0.988, sensitivity: 96.8-98.4%, specificity: 85.5-95.9%. For high myopia (internal test): AUC: 0.978, sensitivity: 91.3%, specificity: 94.5%. For high myopia (external test): AUC: 0.913-0.966, sensitivity: 85.3-97.8%, specificity: 76.4-95.5%.
Du et al. (2021) [9]	China	Pathologic myopia, myopic maculopathy	Feature mining, severe myopic maculopathy classification	457 eyes	CFP	ML with radiomics, random forest	AUC: 0.8263 for new radiomics features, AUC: 0.7925 for clinic features, AUC: 0.8358 for combined features
Lu et al. (2021) [10]	China	Pathologic myopia, myopic maculopathy	Grading, diagnosis, detection, classification	32,010 images	CFP	ResNet-18, Faster R-CNN+FPN	Algorithm I: AUC: 0.995, accuracy: 0.973, sensitivity: 0.939, specificity: 0.981. Algorithm II: macro-AUC: 0.979, accuracy: 0.967, kappa: 0.988. Algorithm III (for “Plus” lesions): accuracy: 0.970-0.994, F1 score: 0.685-0.889
Ye et al. (2021) [11]	China	Myopic maculopathy	Diagnosis, detection, classification	2,342 images	OCT	ResNeSt101	AUC: 92.7-97.4%, sensitivity: 73.9-92.8%, specificity: 84.8-94.0%
Du et al. (2022) [12]	Japan	Myopic traction maculopathy, dome-shaped macula, myopic neovascularization	Diagnosis, grading	9.176 images	SS-OCT	Darknet-19	MTM model: AUC: 0.946, AUPR: 0.876. DSM model: AUC: 0.978, AUPR: 0.653. MNV model: AUC: 0.985, AUPR: 0.908
He et al. (2022) [13]	China	Myopic maculopathy	Classification	3.400 images	OCT	Transfer learning, ResNet-18	Test dataset: AUC: 0.986, accuracy: 96.04%, Kappa: 0.940. External validation dataset: AUC: 0.938, accuracy: 90.6%, Kappa: 0.897
Tang et al. (2022) [14]	China	Pathologic myopia, myopic maculopathy	Diagnosis, grading, segmentation, monitoring of progression	1.395 images	CFP	ResNet-50, DeepLabv3+	Accuracy: 0.9370, sensitivity: 96.67%, specificity: 99.15%, AUC: 0.9980, F1 > 0.8, Kappa: 0.9651
Li et al. (2022) [15]	China	Pathologic myopia, myopic maculopathy, tessellated fundus	Detection, classification	57.148 images	CFP	DCNN-DS, EfficientNet-B0	Internal testing: accuracy: 96.5%, Kappa: 0.922, AUC for PM: 0.997, AUC for TF: 0.985. External testing (SDEH dataset): PM: sensitivity: 93.3%, specificity: 99.6%, AUC: 0.998. TF: sensitivity: 98.8%, specificity: 95.6%, AUC: 0.986. External testing (QDEH Dataset): PM: sensitivity: 91.0%, specificity: 98.7%, AUC: 0.994. TF: sensitivity: 92.8%, specificity: 94.1%, AUC: 0.970
Mao et al. (2023) [16]	China	High myopia, myopic maculopathy	Grading, Analysis of vascular morphological characteristics in high myopia	421 eyes	UWF	Transfer learning, RU-net	Accuracy: 98.24%, sensitivity: 71.42%, specificity: 99.37%, precision: 73.68%, F1 score: 72.29
Wan et al. (2023) [17]	China	Myopic maculopathy	Diagnosis, screening, classification	1.750 images	CFP	VOLO-D2, EfficientNetV2, ResNet50	VOLO-D2 model (best model): accuracy: 96.60%, Kappa: 95.60%
Zhu et al. (2023) [18]	China	Myopic maculopathy	Classification	4.252 images	CFP	Alfa-Mix+ Algorithm, Efficient Former, ResNet18, DenseNet169	Efficient Former + ALFA-Mix+ (best model): accuracy: 0.8964, sensitivity: 0.8643, specificity: 0.9721, Kappa: 0.8537
Zheng et al. (2024) [19]	China	Myopic maculopathy	Diagnosis, grading	1.199 images	CFP	Efficient Net (B0/B7), VGG16, ResNet50	EfficientNet-B0 (best model): sccuracy: 88.32%, Kappa: 83.58%
Chen et al. (2024) [20]	China	Myopic macular degeneration	Prognosis, progression	660 images (internal dataset) 212 images (external dataset)	CFP, SS-OCT	XGBoost	Internal validation dataset: sccuracy: 0.84 ± 0.01, sensitivity: 0.83 ± 0.02, specificity: 0.85 ± 0.01, F1 score: 0.84 ± 0.01, AUROC: 0.84 ± 0.006, AUPRC: 0.87 ± 0.007. External validation dataset: accuracy: 0.80 ± 0.01, sensitivity: 0.97 ± 0.01, specificity: 0.63 ± 0.03, F1 Score: 0.83 ± 0.01, AUROC: 0.80 ± 0.008, AUPRC: 0.83 ± 0.01

Discussion

The analysis of the selected studies provides valuable information regarding the reliability and usefulness of each investigation. An initial review reveals that the studies that included a higher amount of OCT or CFP images were more suitable for the classification, diagnosis, and risk assessment of the disease’s evolution compared to those that were built on a smaller amount of data. This gathering of a larger volume of photographs increases the generalizability of the study and ensures its validity and diversity leading to improved performance. AI models developed by researchers such as Du et al. (2022) [12], Tan et al. [8], He et al. [13], Zhu et al. [18], Lu et al. [10], and Ye et al. [11], who utilized extensive datasets, demonstrated superior performance across parameters such as sensitivity, specificity, accuracy, AUC, and kappa value. By contrast, studies conducted by Chen et al. [20] and Du et al. (2021) [9], which were based on smaller datasets, exhibited relatively lower performance metrics.

Furthermore, the research conducted by Mao et al. [16] introduced the special feature of utilizing more advanced techniques (317 UWF images) and presented the highest accuracy (98.24%) and specificity (99.37%) measurements in relation to the studies cited before, with the drawback of achieving lower sensitivity (71.42%), precision (73.68%), and F1 score (72.29%) results. Moreover, the use of OCT imaging has been shown to be a more accurate diagnostic instrument that allows precise assessment of disease progression compared to CFPs. Nevertheless, it does not constitute the first-choice examination for the screening of these patients since it is more time-consuming and expensive. This observation brings to the surface the need to exploit multimodal imaging (CFP, OCT, and UWF) to function cooperatively in clinical settings for future studies. This proposal will prospectively accomplish a more complete approach and deeper understanding of MM, always including the catalytic role of AI in this endeavor. Another critical point is the limited nationality range (Chinese population) that constitutes a common element in all of the studies analyzed. The exclusion of other ethnicities limits the generalizability of results and detracts from the scalability of research on a multinational and European level. Therefore, future involvement and funding of similar scientific research projects in populations of different countries will unfold the perspective of their potential application on a worldwide scale.

A key conclusion of the analysis is the comparison between human experts and AI in assessing MM lesions. DL algorithms reached the levels of a general ophthalmologist in diagnosing the disease but maintained lower performance compared to senior retina specialists. This observation indicates that the construction of AI models based on DL is still at an experimental stage and needs further processing before reaching full autonomy and replacing the clinical decisions made by experts in this field. However, their utility and helpful role in the initial screening of MM patients and the early detection of sight-threatening complications, such as glaucoma and myopic choroidal neovascularization, is widely acknowledged.

Another crucial aspect in the deployment of AI models for MM assessment is explainability. The ability to interpret how AI arrives at its conclusions is fundamental for its acceptance in clinical practice. The Explainable AI (XAI) techniques, such as Grad-CAM and SHAP used in Ye et al. [11], Wan et al. [17], Chen et al. [20], and other studies, enhance transparency by highlighting the most relevant regions in an image that contribute to the model’s decision. This fosters trust among ophthalmologists and enables validation and refinement of AI predictions, improving overall diagnostic reliability.

In general terms, the technology embodied in AI algorithms can optimize the provision of accurate healthcare services, enhance scientific research on MM screening and identification, and offer a complementary advantage to human expertise. A supplementary element that could strengthen the system’s complexity and improve its modernization index is the integration of additional information such as medical history, demographic data, comorbidities, and genetic indicators of each examined case. This approach could signal a revolutionary phase in data accumulation, image decoding, initial screening, and automatic identification of MM cases, always in cooperation with AI detection systems. Finally, this innovation can optimize disease control strategies by adapting treatment guidelines to each patient’s needs, facilitating in this manner effective monitoring of disease progression, and upgrading the overall quality of life on a broader scale.

Limitations

Each of these studies appeared with several limitations such as restriction to the Japanese population for the one conducted by Du et al. in 2022 [12] (absence of application to other ethnicities for a more objective consideration), use of patients’ images with prior vitreoretinal surgery, macular atrophy not examined, underdiagnosis of controversial cases and need for optimization of the threshold value to balance sensitivity and specificity. Moreover, the researchers included OCT images exclusively (no use of multimodal imaging) in their investigation that are often accompanied by artifacts in MM patients due to increased axial length. This generates errors and noise signals that alter image quality and influence the validity of grading results, thus hindering the effective diagnosis of the entity. The research conducted by Tan et al. [8] included the following drawbacks: detection of MMD as a binary (present or absent) result, not identifying the five categories of the META-PM classification system or individual plus lesions due to the small proportion of these images in the training dataset, narrow spectrum in the detection of myopic findings such as optic nerve head tilt, peripapillary atrophy, posterior staphylomas, and finally low image resolution leading to reduced performance and generalization ability of the algorithm. The investigation carried out by Chen et al. in 2024 [20] using 660 CFPs presented the following limitations: external validation of the model was held in a separate Chinese cohort with a short follow-up period (four years), lack of assessment of its generalizability to other ethnic groups apart from the Chinese population sample, influence of the COVID-19 pandemic in scheduling the visits for ophthalmological examination, utilization of the SS-OCT diagnostic tool alone (no SD-OCT assessment), insufficient depiction of posterior staphyloma using 45° fundus camera (limited imaging range), and need for utilization of other imaging techniques for a more holistic evaluation and therefore a more precise diagnosis.

The study published by Mao et al. in 2023 [16], which included UWF images of 421 eyes, was a single-center, hospital-based research and a vague peripheral deformation of the photographs was noted. In addition, Wan et al. in 2023 [17] used a moderate amount of data (1,750 CFPs) due to difficulty in collection, leading to an incorrect diagnosis of some lesions and highlighting the need for improvement in image segmentation methods. He et al. in 2022 [13] evaluated a larger amount of 3,400 OCT images to classify MM. Nevertheless, a low quality of the images captured was observed and a co-existence of multiple types of MTM in one picture of each case. Du et al. in 2021 [9] explored the field of pathologic myopia to arrive at an MM classification (severe stage) using ML by analyzing CFPs from 457 eyes. However, the absence of follow-up data for further testing of the hypothesis was a significant drawback of this study. The research carried out by Zhu et al. in 2023 [18] regarding the deployment of a grading system for high MM cases with the contribution of a DL algorithm and the utilization of a great amount of color retinal fundus images (4,252) exhibited limitations in the following domains: scalability, computational efficiency, image segmentation, and quality of the samples used. Two years before (2021), Lu et al. [10] examined a great amount of 32,010 CFPs to grade MM using CNN as a reference algorithm. Even though the model achieved satisfactory performance, the need for multimodal imaging (OCT, OCTA, and FA) was emphasized for more successful detection of posterior staphylomas and lacquer cracks. During the same year, Ye et al. [11] screened and classified 2,342 OCT images with the assistance of a DL AI system that raised the following issues: miscalculation, low image quality, low accuracy, misdiagnosis, and only two medical centers involved (need for multicenter research). The next year (2022), Tang et al. [14] and Jun Li et al. [15] focused their research on CFP analysis to diagnose MM via the exploitation of DL tools. Despite the algorithms’ fulfillment in achieving adequate evaluation metrics, some of the indicative disadvantages are listed below: small quantity of images, reduced generalization ability of the model absence of OCT imaging data in the case of the aforementioned authors, and simplification of the META-PM classification system, absence of OCT and wide-field fundus imaging, lack of automatic image quality evaluation and assessment of clinical samples only (community population examples not included in investigation) as for the editors mentioned second. Finally, Zheng et al.’s [19] research in 2024 on an AI-based MM grading method using EfficientNet presented with the following limitations: no inclusion of OCT images that could offer more detailed information on anatomical structures of the macular region (collection of CFFs exclusively), since the combination of different imaging modalities could yield better results. In addition, the models trained for recognition and diagnosis of the disease are challenging to apply in real-world settings, whereas restricted DCA (diffuse chiorioretinal atrophy) and PCA (patchy chorioretinal atrophy) data led to lower sensitivities of 75.98% and 64.67%, respectively. These results are better suited for preliminary disease screening rather than substituting professional diagnoses made by specialists in this field.

Although XAI techniques help make AI decisions more understandable, it is not always reliable. The way AI highlights important features can be inconsistent, which may lead to confusion for doctors. More research is needed to make these explanations clearer and ensure they are truly helpful in medical practice.

The issue of cost-effectiveness should not be neglected, especially when it comes to the application of dual-stream D-CNN models as proposed by Li et al. in 2022 [15], whose evaluation of feasibility in a real-world setting needs further investigation. In addition, the inclusion of the OCT imaging modality (especially enhanced depth imaging (EDI)) that offers the supplementary advantage of fovea choroidal thickness calculation along with the basic fundus photograph examination is a crucial procedure that needs to be implemented on the first screening of these patients. On the other hand, the additional utilization of more advanced imaging techniques such as wide-field fundus imaging is imperative to permit assessment of the periphery of each highly myopic fundus and to primarily detect alterations attributed to increased axial length in this high-risk population. Lastly, an independent quality control check should be performed in each of the studies mentioned above, since the existence of ineligible images due to non-clear refractive media (e.g. cataract, prior vitreoretinal surgery) limits the statistical reliability of investigations.

Finally, the observations cited above indicate that a more careful and rational selection of samples from the general population is necessary to improve the generalizability of the models exploited and thus increase the objectivity index and eliminate to a significant extent the systematic errors of the studies examined. Following this direction, complementary collaboration between centers on a worldwide scale should be promoted to combat the lack of diversity and achieve high-quality dataset collection amongst different population groups. Undoubtedly, ethical issues regarding transparency, human bias, data protection, decision making, and liability need to be addressed, and global barriers must be crossed before the final legalization of the methodologies analyzed in the previous part. Despite these challenges, AI, ML, and DL algorithms are aspired to have a significant clinical impact on MM recognition in the years to come. However, while AI models perform at a level comparable to general ophthalmologists, their lower accuracy compared to senior retina specialists highlights a key limitation, reinforcing the need for further improvements before full clinical adoption.

## Conclusions

The investigations conducted so far in this field show that AI-based models can be successfully applied to the initial screening of MM patients on a large scale in the future, reducing the workload of ophthalmologists and improving the techniques of immediate intervention when major, sight-threatening complications arise. The question of whether experts’ evaluation and critical thinking can be replaced by automated analysis techniques remains unanswered, since it constitutes a controversial issue that requires extensive discussion to be resolved. In every situation, the studies mentioned above prove that the application of deep learning and machine learning methodologies that are based on the construction of convolutional and artificial neural networks may play a supportive role and function as catalysts in the management of MM cases in everyday clinical practice. The scientists involved in these studies successfully arrived at valid conclusions using statistical measurements regarding the effectiveness of various AI models in the diagnosis, classification, initial screening, and follow-up of highly myopic cases. However, the need for the application of more advanced and diverse imaging techniques remains imperative to improve image quality and to achieve a more holistic consideration and thorough assessment of this patient population.

## References

[1] Ruiz-Medrano J, Montero JA, Flores-Moreno I, Arias L, García-Layana A, Ruiz-Moreno JM (2019). Myopic maculopathy: current status and proposal for a new classification and grading system (ATN). Prog Retin Eye Res.

[2] Müller D, Soto-Rey I, Kramer F (2022). An analysis on ensemble learning optimized medical image classification with deep convolutional neural networks. IEEE Access.

[3] Hayashi K, Ohno-Matsui K, Shimada N (2010). Long-term pattern of progression of myopic maculopathy: a natural history study. Ophthalmology.

[4] Yokoi T, Ohno-Matsui K (2018). Diagnosis and treatment of myopic maculopathy. Asia Pac J Ophthalmol (Phila).

[5] Ohno-Matsui K, Kawasaki R, Jonas JB (2015). International photographic classification and grading system for myopic maculopathy. Am J Ophthalmol.

[6] Frisina R, Gius I, Palmieri M, Finzi A, Tozzi L, Parolini B (2020). Myopic traction maculopathy: Diagnostic and management strategies. Clin Ophthalmol.

[7] Page MJ, McKenzie JE, Bossuyt PM (2021). The PRISMA 2020 statement: an updated guideline for reporting systematic reviews. BMJ.

[8] Tan TE, Anees A, Chen C (2021). Retinal photograph-based deep learning algorithms for myopia and a blockchain platform to facilitate artificial intelligence medical research: a retrospective multicohort study. Lancet Digit Health.

[9] Du Y, Chen Q, Fan Y (2021). Automatic identification of myopic maculopathy related imaging features in optic disc region via machine learning methods. J Transl Med.

[10] Lu L, Ren P, Tang X (2021). AI-model for identifying pathologic myopia based on deep learning algorithms of myopic maculopathy classification and “plus” lesion detection in fundus images. Front Cell Dev Biol.

[11] Ye X, Wang J, Chen Y (2021). Automatic screening and identifying myopic maculopathy on optical coherence tomography images using deep learning. Transl Vis Sci Technol.

[12] Du R, Xie S, Fang Y (2022). Validation of soft labels in developing deep learning algorithms for detecting lesions of myopic maculopathy from optical coherence tomographic images. Asia Pac J Ophthalmol (Phila).

[13] He X, Ren P, Lu L, Tang X, Wang J, Yang Z, Han W (2022). Development of a deep learning algorithm for myopic maculopathy classification based on OCT images using transfer learning. Front Public Health.

[14] Tang J, Yuan M, Tian K (2022). An artificial intelligence based automated grading and lesions segmentation system for myopic maculopathy based on color fundus photographs. Transl Vis Sci Technol.

[15] Li J, Wang L, Gao Y (2022). Automated detection of myopic maculopathy from color fundus photographs using deep convolutional neural networks. Eye Vis (Lond).

[16] Mao J, Deng X, Ye Y (2022). Morphological characteristics of retinal vessels in eyes with high myopia: ultra-wide field images analyzed by artificial intelligence using a transfer learning system. Front Med (Lausanne).

[17] Wan C, Fang J, Hua X, Chen L, Zhang S, Yang W (2023). Automated detection of myopic maculopathy using five-category models based on vision outlooker for visual recognition. Front Comput Neurosci.

[18] Zhu SJ, Zhan HD, Wu MN, Zheng B, Liu BQ, Zhang SC, Yang WH (2023). Research on classification method of high myopic maculopathy based on retinal fundus images and optimized ALFA-Mix active learning algorithm. Int J Ophthalmol.

[19] Zheng B, Zhang M, Zhu S, Wu M, Chen L, Zhang S, Yang W (2024). Research on an artificial intelligence-based myopic maculopathy grading method using EfficientNet. Indian J Ophthalmol.

[20] Chen Y, Yang S, Liu R (2024). Forecasting myopic maculopathy risk over a decade: development and validation of an interpretable machine learning algorithm. Invest Ophthalmol Vis Sci.

